# Recurrent/metastatic relapse after definitive treatment for HNSCC: timing, patterns, and survival

**DOI:** 10.3389/fonc.2026.1738860

**Published:** 2026-03-11

**Authors:** Kewen Qu, Margaret Stalker, Wei-Ting Hwang, Roger B. Cohen, Lova Sun

**Affiliations:** 1 Department of Biostatistics, Epidemiology, and Informatics, Perelman School of Medicine, University of Pennsylvania, Philadelphia, PA, United States; 2 Department of Medicine, Perelman School of Medicine, Perelman School of Medicine, University of Pennsylvania, Philadelphia, PA, United States; 3 Division of Hematology & Oncology, Perelman School of Medicine, University of Pennsylvania, Philadelphia, PA, United States

**Keywords:** definitive therapy, recurrent/metastatic relapse, recurrent/metastatic systemic therapy, chemoradiation (CRT), trimodality therapy, head and neck squamous cell carcinoma

## Abstract

**Background:**

Timing and outcomes of recurrence after definitive therapy for HNSCC are incompletely understood.

**Methods:**

This study included patients in a nationwide EHR-derived de-identified database who received systemic therapy for R/M HNSCC. Time from definitive treatment to R/M systemic therapy initiation (TTRM), and overall survival (OS) from R/M systemic therapy initiation, were estimated and compared between patients stratified by cancer site and prior definitive treatment modality.

**Results:**

Of 7657 patients receiving R/M therapy, the median age was 65 (IQR 58-72), 77% male, 74% white, 79% with smoking history, and 79% ECOG PS 0-1. 1684 (22%) patients had no recorded prior definitive therapy (*de novo* metastatic); of the remaining 5973 patients, most common definitive treatment types were radiation alone (RT only; 43%), followed by surgery plus radiation (Surg + RT; 21%), surgery alone (Surg only; 16%), chemoradiation (CRT; 14%), and surgery plus chemoradiation (Surg + CRT; 6%). Patients treated with high-risk treatment types (Surg + CRT, CRT) had higher disease stage at presentation, shorter TTRM (median 6–7 vs 17–19 months), and shorter OS from R/M treatment to death (median 8 vs 12 months) compared to patients in low-risk treatment groups (RT only, Surg + RT, Surg only). Patients with HPV+ oropharynx cancer had numerically longer TTRM (median 17 vs 13 months, p = 0.4309) and significantly longer OS (median 18 vs 10 months, p < 0.001) than HPV- cancers.

**Conclusions:**

Patients with locoregionally advanced HNSCC treated with CRT and surgery plus CRT represent a high-risk cohort with rapid R/M disease and poor survival, who need improved therapeutic options.

## Introduction

Head and Neck Squamous Cell Carcinoma (HNSCC) is a heterogeneous disease associated with persistently high morbidity and mortality (1, 2). Most HNSCCs are non-metastatic (M0) at diagnosis, and confined to the primary site with or without involvement of regional lymph nodes (3). Standard treatment for locoregional HNSCC is either surgery followed by adjuvant (chemo)radiation as dictated by pathologic features, or definitive (chemo)radiation. Management varies, however, based on stage, location of the cancer, functional status, and other patient and disease-specific factors (4).

Despite definitive treatment with curative intent, approximately 15-50% of patients will develop recurrent or metastatic (R/M) disease (5, 6), which occurs within 6 months (rapid recurrences) after definitive therapy in about half of patients (7). Recurrence is most commonly locoregional, but distant metastases alone occur in about a quarter of patients (8, 9). Patients with R/M HNSCC have a generally poor prognosis with median overall survival of about a year (10–12), even with contemporary standard of care immunotherapy (13). Our understanding of timing and patterns of relapse after definitive therapy, and the impact of factors including disease site and primary treatment modality, remains limited. To address this knowledge gap, we conducted a nationwide observational study of patients with R/M HNSCC aimed at describing timing of recurrence from initial definitive-intent therapy, as well as overall survival.

## Methods

### Study sample and subsets

We included adult patients who received systemic treatment for mucosal R/M HNSCC (oropharynx, larynx, oral cavity, hypopharynx, or unknown primary site) from the Flatiron Health electronic medical record (EHR)-derived de-identified electronic medical records from 01/13/2011-09/28/2023. The Flatiron Health database is a longitudinal database, comprising deidentified patient-level structured and unstructured data, curated via technology-enabled abstraction (14, 15). During the study period, the deidentified data originated from approximately 280 US cancer clinics (approximately 800 sites of care). The data are deidentified and subject to obligations to prevent re-identification and protect patient confidentiality. As previously described (16), frontline R/M systemic therapy (1L), intended to capture initial systemic treatment for recurrent/metastatic disease, was defined as systemic therapy starting ≥ 60 days after any recorded radiation to the primary site, on order to avoid capturing chemoradiotherapy in the definitive setting.

### Study measures

Patients’ initial definitive treatments (pre-1L) were captured from the database. All patients’ definitive treatment was categorized as surgery plus chemoradiation (trimodality) (Surg + CRT), chemoradiation (CRT), surgery alone (Surg only), surgery plus radiation (Surg + RT), radiation alone (RT only), or none. Patients were also characterized based on their primary cancer site (HPV+ oropharynx, HPV- oropharynx, or other).

For patients with recorded pre-1L definitive treatment, time from definitive treatment (index date) to initiation of R/M systemic therapy (TTRM) was calculated for each patient. For patients who had surgery, surgical date was used as the index date. For the chemoradiotherapy cohort, the start date of pre-1L systemic therapy was used as the index date. For the radiation only cohort, radiation start date was used as the index date (17).

### Statistical analysis

#### Clinical and demographic characteristics

Descriptive statistics were used to analyze demographic characteristics patients in the total cohort and sub-cohorts, including cohorts by definitive treatment type (Surg + CRT, CRT, Surg + RT, Surg only, and RT only) and cohorts by primary disease sites (HPV+ OP, HPV- OP, and other). Continuous variables (age and socioeconomic status (SES), a 5-level indicator of neighborhood socioeconomic conditions with 1 - lowest SES; 5 - highest SES (18, 19)) were analyzed by median and inter-quartile range (IQR) and categorical variables (sex, race, smoking status, PD-L1 level, ECOG performance status (PS), primary cancer site, pattern (sites) of recurrence, year of treatment, practice type, and type of frontline treatment) were reported through frequency and percentages. A Venn diagram was used to describe the breakdown of pre-1L systemic, radiation, and surgery and any overlaps.

#### Distribution of definitive treatment type by cancer stage at diagnosis

Surg + CRT and CRT were defined as “high-risk” treatment modalities whereas Surg only, Surg + RT, and RT only were defined as “low-risk” treatment modalities, in line with clinical practice and guidelines recommending trimodality therapy and CRT in more locoregionally advanced cases (20). To validate the hypothesis that CRT and trimodality therapy were more commonly used in patients with higher risk and higher stage disease, we used Pearson’s Chi-Square test for trend to investigate the association between clinical stage and definitive treatment type. A bar plot with frequencies and percentages for definitive treatments by different cancer stages were also used to visualize this association.

#### Distribution of time from definitive treatment to R/M treatment

Distribution of time from definitive treatment to R/M systemic (TTRM) treatment was summarized and displayed graphically through bar plots with marginal histograms, by definitive therapy type and by primary cancer sites. Categorized TTRM was also examined graphically using frequencies and fractions of the total. Log-rank tests were used to compare TTRM between groups defined by above mentioned categorical variables, as well as demographics including gender, geographical region, smoking status, socioeconomic status, race, and ECOG performance status.

#### Analysis of survival time from R/M treatment start to death

Kaplan-Meier Curves were used to examine the overall survival (OS) from R/M treatment initiation to death. Median OS by initial definitive treatment type and by primary disease sites were reported separately. Log rank test was used to compare OS between groups, specifically between patients treated with high-risk vs low-risk treatment modalities, high-risk treatment vs *de novo* metastatic disease, HPV+ OP vs HPV- OP cancer, and HPV+ OP vs other primary cancers.

All statistical tests were conducted using STATA v15.0 (State College, TX). P-values of 0.05 were considered significant. This study was granted waiver of informed consent by the institutional review board of the University of Pennsylvania.

### Institutional review board statement

Institutional waiver of consent was granted by the University of Pennsylvania IRB.

## Results

### Total cohort characteristics and primary tumor type

**Figure 1:**
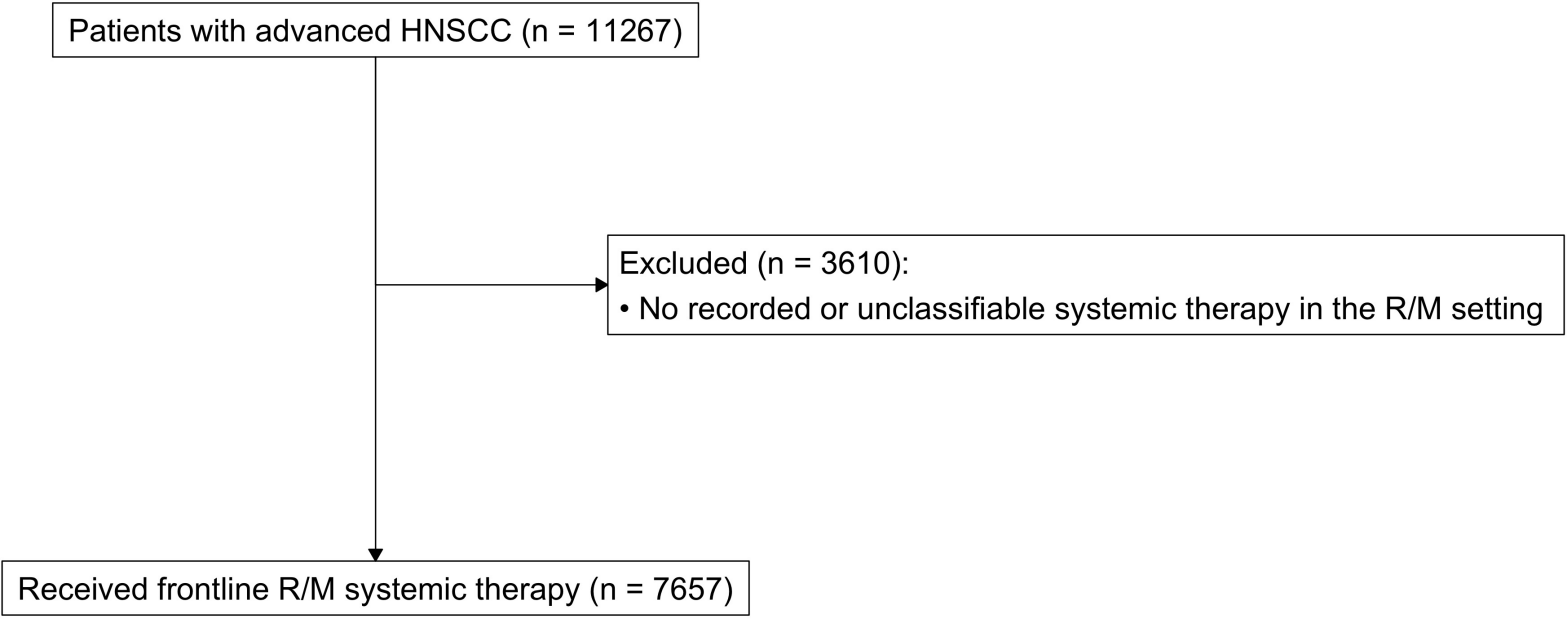
CONSORT diagram.

Among 11267 patients with advanced HNSCC, we included 7657 adult patients who received frontline systemic therapy for R/M HNSCC (**Figure 1****).** Median (IQR) age was 65 (58-72), 77% patients were male, 74% were White, 79% had a smoking history, 79% were ECOG 0-1, 76% were treated in a community setting (**Table 1**). Of patients with recorded PD-L1 status (n = 2260, 30%), distribution of CPS < 1, 1-19, and ≥ 20 was 22%, 41%, and 38%, respectively. Most common primary tumor types were HPV+ oropharynx (n = 1928, 26%), HPV- oropharynx (n = 1581, 21%), oral cavity (n = 1899, 25%) and larynx (n = 1567, 21%). Patients with HPV+ OP cancers had a higher proportion of ECOG PS 0-1 (86% vs 77%/77% in HPV- OP/other), and higher proportion of never smokers (31% vs 14%/20% in HPV- OP/other).

**Table 1:** Demographics for patients of the full cohort and cohorts by primary cancer site.

	Total	HPV+ OPSCC	HPV- OPSCC	Other primary sites^d^
	N=7,657	N=1,928	N=1,581	N=4,148
Age	65 (58-72)	64 (58-71)	65 (58-71)	66 (58-73)
Sex				
Female	1,728 (22.6%)	193 (10.0%)	359 (22.7%)	1,176 (28.4%)
Male	5,929 (77.4%)	1,735 (90.0%)	1,222 (77.3%)	2,972 (71.6%)
Race				
White	5,130 (73.9%)	1,369 (77.7%)	1,038 (72.0%)	2,723 (72.8%)
Black/AA	482 (6.9%)	71 (4.0%)	121 (8.4%)	290 (7.8%)
Asian	96 (1.4%)	1 (0.1%)	20 (1.4%)	75 (2.0%)
Other	1,234 (17.8%)	321 (18.2%)	263 (18.2%)	650 (17.4%)
Missing	715 (-)	166 (-)	139 (-)	410 (-)
Smoking status				
No smoking history	1,639 (21.5%)	597 (31.0%)	218 (13.9%)	824 (20.0%)
Current or former smoker	5,983 (78.5%)	1,327 (69.0%)	1,355 (86.1%)	3,301 (80.0%)
Missing	35 (-)	4 (-)	8 (-)	23 (-)
PD-L1 level				
CPS ≥ 20	852 (37.7%)	259 (34.6%)	116 (33.7%)	477 (40.8%)
CPS 1–19^a^	921 (40.8%)	327 (43.7%)	135 (39.2%)	459 (39.3%)
CPS <1	487 (21.5%)	162 (21.7%)	93 (27.0%)	232 (19.9%)
Unknown^b^	5,397 (-)	1,180 (-)	1,237 (-)	2,980 (-)
ECOG PS				
0-1	5,029 (79.4%)	1,430 (85.9%)	974 (76.5%)	2,625 (77.3%)
≥ 2	1,304 (20.6%)	234 (14.1%)	299 (23.5%)	771 (22.7%)
Missing	1,324 (-)	264 (-)	308 (-)	752 (-)
Definitive treatment				
Surg + CRT	374 (4.9%)	50 (2.6%)	55 (3.5%)	269 (6.5%)
CRT	853 (11.1%)	233 (12.1%)	244 (15.4%)	376 (9.1%)
Surg only	940 (12.3%)	79 (4.1%)	96 (6.1%)	765 (18.4%)
Surg + RT	1,253 (16.4%)	248 (12.9%)	185 (11.7%)	820 (19.8%)
RT only	2,553 (33.3%)	890 (46.2%)	561 (35.5%)	1,102 (26.6%)
No defn tx	1,684 (22.0%)	428 (22.2%)	440 (27.8%)	816 (19.7%)
Patterns of recurrence				
Local only	1,473 (27.1%)	232 (16.2%)	303 (29.6%)	938 (31.5%)
Distant only	3,198 (58.9%)	1,059 (74.0%)	602 (58.9%)	1,537 (51.6%)
Local + distant	762 (14.0%)	141 (9.8%)	117 (11.4%)	504 (16.9%)
Not recorded	2,224 (-)	496 (-)	559 (-)	1,169 (-)
Year of treatment				
2011-2018	4,262 (55.7%)	877 (45.5%)	1,038 (65.7%)	2,347 (56.6%)
2019	765 (10.0%)	225 (11.7%)	126 (8.0%)	414 (10.0%)
2020	735 (9.6%)	224 (11.6%)	126 (8.0%)	385 (9.3%)
2021	736 (9.6%)	223 (11.6%)	125 (7.9%)	388 (9.4%)
2022	725 (9.5%)	236 (12.2%)	112 (7.1%)	377 (9.1%)
2023	434 (5.7%)	143 (7.4%)	54 (3.4%)	237 (5.7%)
SES^c^	3 (2-4)	3 (2-4)	3 (2-4)	3 (2-4)
Practice type				
Community	5,838 (76.2%)	1,426 (74.0%)	1,301 (82.3%)	3,111 (75.0%)
Academic	1,819 (23.8%)	502 (26.0%)	280 (17.7%)	1,037 (25.0%)
Frontline treatment				
Chemotherapy +/- cetuximab	4,675 (61.1%)	1,011 (52.4%)	1,048 (66.3%)	2,616 (63.1%)
CPI with chemotherapy	864 (11.3%)	266 (13.8%)	139 (8.8%)	459 (11.1%)
CPI monotherapy	2,118 (27.7%)	651 (33.8%)	394 (24.9%)	1,073 (25.9%)

Data are presented as median (IQR) for continuous measures, and n (%) for categorical measures.

For categorical variables which include missing/unknown, percentages are out of known categories and missing/unknown are listed without %.

^a^CPS 1–19 includes “positive, not otherwise specified” in 41, 16, 8, and 17 patients with total, HPV+ Oropharynx, HPV- Oropharynx, and other primary sites groups, respectively.

^b^Unknown includes 200, 64, 39, and 97 patients with total, HPV+ Oropharynx, HPV- Oropharynx, and other primary sites groups, respectively, with PD-L1 test attempted but no result available.

^c^5-level indicator of neighborhood socioeconomic conditions (1 - lowest SES; 5 - highest SES).

^d^Other primary sites include 1567, 1899, 487, and 195 patients with larynx, oral cavity, hypopharynx, and unknown primary sites, respectively.

OPSCC, Oropharyngeal squamous cell carcinoma; CPS, combined positive score; ECOG PS, ECOG performance status; Surg, surgery; CRT, chemoradiation; RT, radiation; defn tx, definitive treatment; SES, socioeconomical status.

### Prior definitive therapy modalities

**Figure 2:**
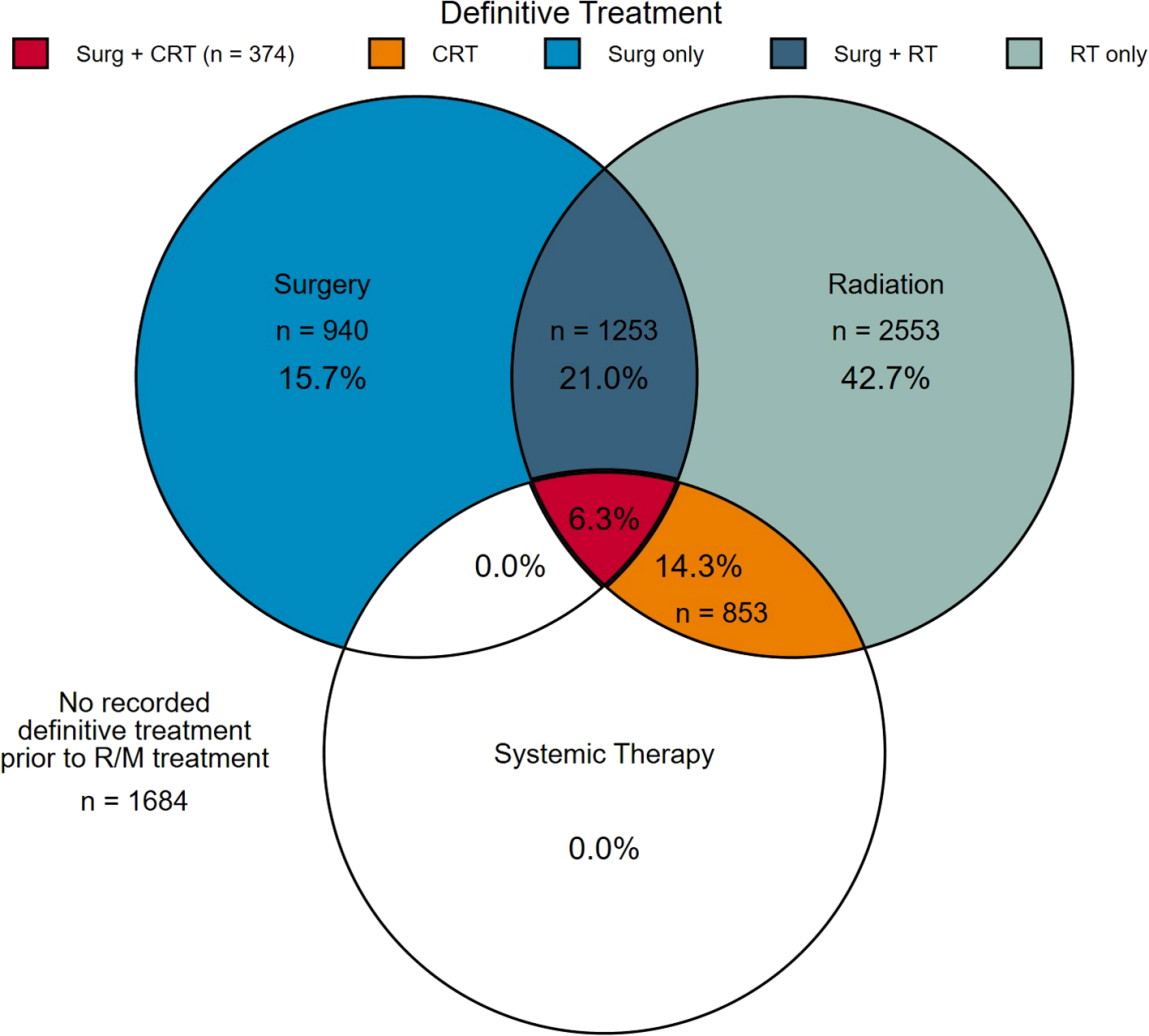
Receipt of definitive radiation, surgery, and/or systemic therapy, or none before systemic therapy for recurrent/metastatic disease (n=7657).

Within the total cohort of 7657 patients, 78% (n = 5973) patients had prior definitive treatment, whereas 22% (n = 1684) had no recorded prior definitive treatment. Among patients with recorded prior definitive treatment, 940 (16%) had surgery alone, 1253 (21%) had surgery followed by RT, and 374 (6%) had surgery followed by CRT (trimodality); 2553 (43%) had RT alone, and 853 (14%) had CRT (**Figure 2**).

Over two thirds of patients in our cohort had stage 3 (n = 973, 19%) or stage 4 (n = 2956, 59%) disease at diagnosis, reflecting a cohort with mostly locally advanced disease who went on to experience recurrent/metastatic disease. As expected, patients with higher cancer stage at diagnosis were more commonly treated with high-risk treatment types (CRT and trimodality) (**Supplementary Figure 1**). Risk of the selected definitive treatment type increased linearly with the cancer stage at diagnosis, Pearson’s Chi-Square p-value < 0.001.

Most other baseline characteristics did not differ by definitive treatment type (**Supplementary Table 1****).** Notably, performance status did not significantly differ between treatment approaches, with 75% to 81% of patients in all treatment types with recorded PS having ECOG PS 0-1. Unsurprisingly, patients with oropharynx cancers made up the majority of patients treated with primary CRT (58%) and RT alone (58%), whereas those with oral cavity cancers accounted for the majority of Surg only (68%), Surg + RT (44%), and Surg + CRT (55%).

### Recurrence patterns

Of patients with a recorded recurrence pattern (n = 5,433, 71%), 27% patients had local/regional recurrence only, 59% patients had distant recurrence only, and 14% patients had both local and distant recurrence (**Table 1**). Of patients with HPV+ OP cancer with a recorded recurrence pattern (n = 1432, 74%), the majority (74%) had distant recurrence only, compared to 59% with HPV- OP and 52% with other primary sites. Interestingly, while almost all patients treated with high-risk modalities had distant only disease recurrence (98% for Surg + CRT and 99% for CRT), a higher proportion of patients treated with low-risk modalities experienced local recurrence, with or without distant recurrence (75%, 45%, and 41% for Surg only, Surg + RT, and RT only, respectively).

### Months from definitive treatment to R/M treatment by type of definitive treatment, primary cancer site, and key demographics

**Figure 3:**
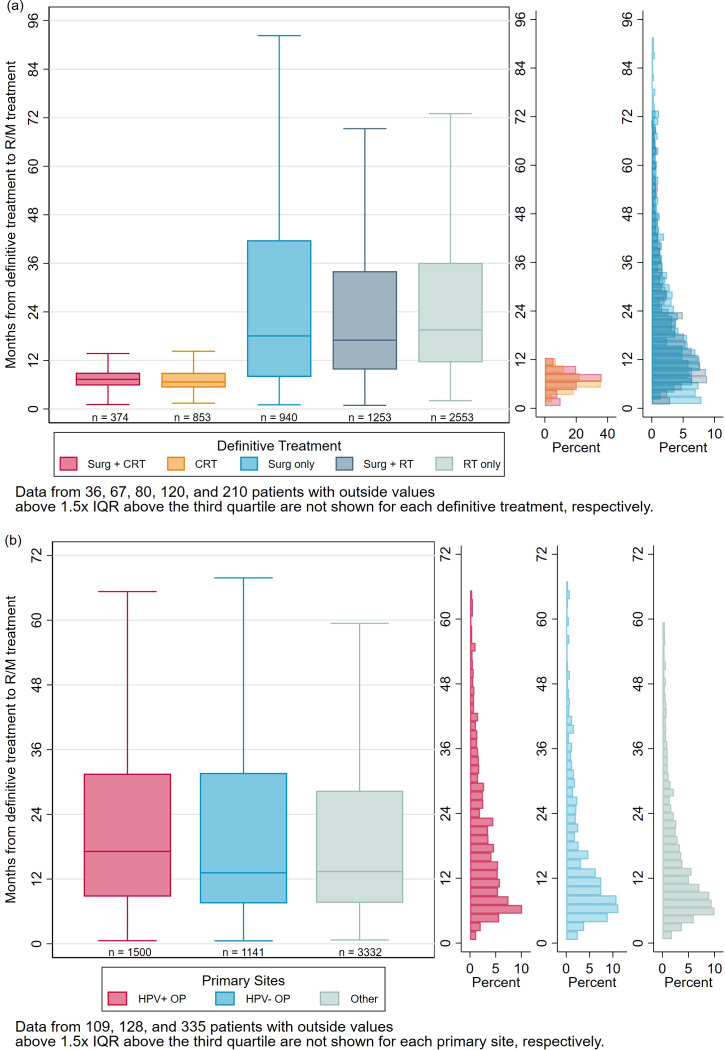
Marginal Histogram/Box plot for TTRM by **(a)** Definitive Treatment Type and **(b)** Disease site.

The median (IQR) of time from definitive treatment to R/M treatment (TTRM) was 14 (8-30) months for the whole cohort. Patients treated with surgery + CRT and CRT had significantly shorter TTRM (median 7 and 7 months, respectively) compared to patients treated with surgery only, surgery + RT, and RT only (median 18, 17, and 20 months, respectively) (**Figure 3a**). High-risk treatment categories had significantly shorter TTRM than low-risk treatment categories (log-rank p < 0.001). Almost all patients treated with Surg + CRT (98%) and CRT (99%) started R/M treatment within 24 months of definitive treatment, compared to 61%, 65%, and 61% of patients treated with Surg only, Surg + RT, and RT only, respectively. (**Supplementary Figure 2****).** Rapid recurrence within 6 months occurred in 42% and 55% of the Surg + CRT and CRT groups, compared to only 22%, 13%, and 9% of the lower-risk treatment groups, respectively.

Differences in recurrence timing by HPV status and primary site was also explored (**Figure 3b**). Although TTRM was numerically longer in HPV+ OP (median 17 months, IQR 9-32), than HPV- OP (13 months, IQR 8-32) and other primary site (13 months, IQR 8-28) cancers, this difference was not statistically significant (log-rank p = 0.4309). Median TTRM was 11 months in hypopharynx cancers, 12 months in oral cavity cancers, and 14 months in larynx cancers.

There was no significant difference in TTRM by gender (log-rank p = 0.7019), geographical region (log-rank p = 0.5304), academic vs community practice type (log-rank p = 0.3705), SES (log-rank p = 0.1910), or race (log-rank p = 0.1732). Shorter TTRM was observed in patients with smoking history compared to those without smoking history (median 14 vs 15 months; log-rank p = 0.0442) and in patients with ECOG ≥ 2 compared to those with ECOG 0-1 (median 11 vs 15 months; log-rank p = 0.0003).

### Overall survival from R/M treatment to death by definitive treatment type and primary cancer site

**Figure 4:**
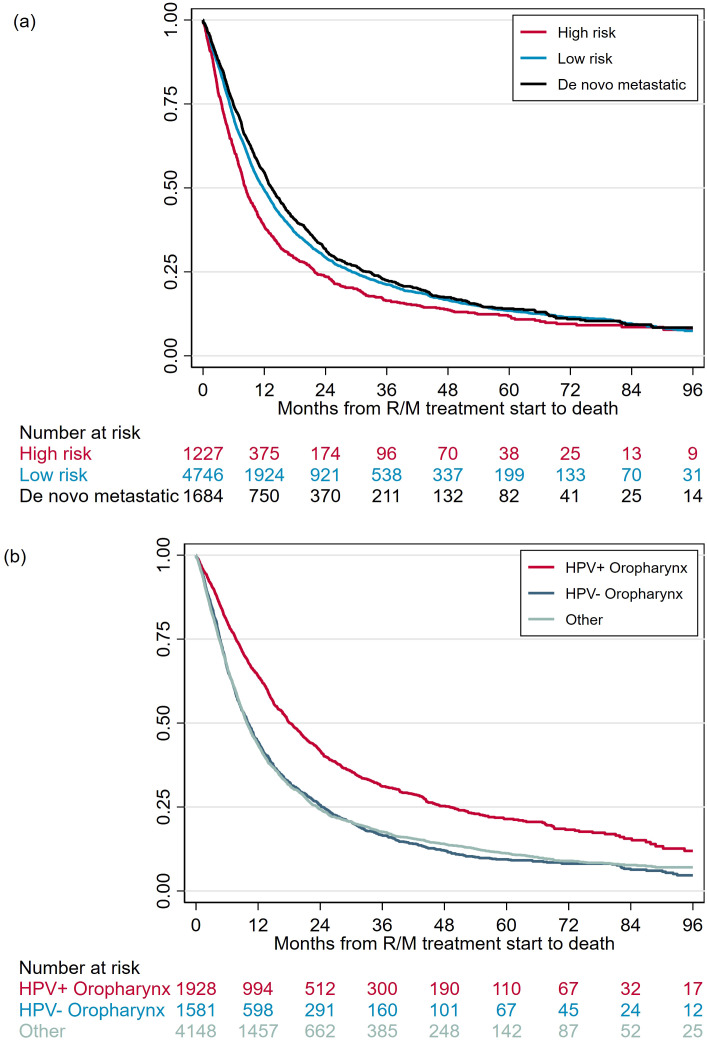
Kaplan-Meier Curves for OS by **(a)** definitive treatment type and **(b)** primary disease site.

The median (IQR) overall survival from R/M treatment initiation was 12 (5-28) months for the overall cohort. OS was significantly shorter for patients previously treated with high-risk definitive modalities (median 8 months; IQR 4-22) compared to with low-risk modalities (median 12 months; IQR 5-29, log-rank p < 0.001), as well as compared to patients with *de novo* metastatic disease (median 13 months; IQR 6-33, log-rank p < 0.001) (**Figure 4a**). Among patients treated with low-risk modalities, OS did not differ significantly by recurrence pattern: median OS was 11, 12, and 13 months in patients with local, distant, and local + distant recurrence, respectively.

Survival was longer in patients with HPV+ OPSCC patients (median 18 months; IQR 8-49) compared to HPV- OPSCC (median 10 months; IQR 5-24, log-rank p < 0.001) as well as compared to other cancers (median 10 months; IQR 5-23, log-rank p < 0.001) (**Figure 4b**). Median OS was 11 months for hypopharynx and larynx cancers, and 9 months for oral cavity cancers.

In total, 47% of patients had documented second line R/M treatment. This proportion was higher in patients with *de novo* metastatic disease (53%) than those with recorded definitive therapy (RT, 48%; surgery, 43%; surgery + RT, 45%; CRT, 43%; surgery + CRT, 44%).

## Conclusions

In this US nationwide study of over 7,500 patients who received systemic therapy for R/M HNSCC, most recurrences occurred within 2 years of definitive therapy (median TTRM 14 months). Compared to patients with lower-stage disease, patients with higher-stage disease were more commonly treated with and CRT and trimodality therapy, so-called high-risk treatment modalities, as opposed to surgery or radiation alone, or surgery with adjuvant radiation. Unsurprisingly, these patients treated with high-risk modalities had a dramatically shorter time to R/M treatment (median 6–7 vs. 17–19 months), as well as a significantly shorter subsequent OS after initiation of R/M treatment (8 vs 12 months), compared to those treated with lower risk treatment modalities. Interestingly, patients without recorded definitive therapy (i.e., *de novo* metastatic disease) had similar survival to those treated with lower-risk treatment types.

This finding is not attributable to differential distribution of treatment types across primary cancer types, as (1) difference in TTRM among primary tumor sites was much less dramatic than among treatment types, and (2) the split between high- and low-risk treatment modalities was similar across disease types, as high-risk oropharynx cancers preferentially underwent CRT while high-risk oral cavity cancers preferentially received trimodality therapy. The marked discrepancy in TTRM by definitive treatment type does not indicate a causal link between intensive therapy and rapid recurrence, but rather serves as a marker of disease aggressiveness and underscores that high-risk locoregionally advanced patients, despite undergoing more intensive curative-intent definitive therapy, have rapid recurrences and poor survival outcomes.

HPV+ cancers had numerically longer time to recurrent/metastatic relapse than HPV- cancers (median 17 vs 13 months) (21–23), as well as longer OS after R/M therapy initiation, similar to prior reports (24, 25). In patients with HPV+ OPSCC in our cohort, nearly three quarters had distant recurrence only, in contrast to a more balanced mix of locoregional and distant recurrences in HPV- cancers. This finding differs somewhat from prior smaller studies showing no difference in patterns of recurrence by HPV status (22, 26, 27), although several reports have shown later and more disseminated metastatic involvement with HPV+ compared to HPV- cancers (7, 28). Our results add to the literature and clinical experience showing that HPV+ OPSCC exhibits a distinct natural history, both in timing and patterns of recurrence, as well as a well-documented favorable prognosis compared to HPV- cancers (29).

Our findings have important implications for clinical trial study design. Most clinical trials of systemic therapy in the frontline R/M setting, including the pivotal KN-048 and EXTREME trials, excluded patients who received systemic therapy in the definitive setting within 6 months (8, 13, 30). In our cohort, 50% of patients in high-risk treatment groups recurred within 6 months of prior definitive therapy, and would automatically be excluded from clinical trial participation. The resulting skewed representation (i.e. preferential inclusion of patients in trials with lower-risk and more indolent disease) may lead to crucial biases in conclusions regarding efficacy and safety of approved and novel agents. This and other restrictive clinical trial exclusion criteria likely lead to optimistically biased results, as well as an ongoing inability to study, understand, and appropriately counsel the large population of patients with more aggressive disease biology.

Limitations of our retrospective analysis include incomplete data capture of variables such as recurrence pattern, cancer stage, and PD-L1 expression. In particular, missingness in the recurrence pattern variable was high, and non-standard data capture may have accounted for some of the differences between locoregional and distant recurrence between categories, so limited conclusions can be drawn from this analysis. AJCC staging system was not always recorded in the database, so cancer staging information was not uniform across patients. Some patients with prior uncaptured definitive therapy performed at an outside institution may have been miscategorized as *de novo* metastatic disease, although this represented a relatively small proportion of our cohort. Because this analysis included only patients treated for recurrent/metastatic disease (due to characteristics of the database which focused on advanced/metastatic disease), the true denominator of patients receiving definitive treatment is unknown, and thus we were not able to comment on rates of recurrence by treatment type. Finally, as previously mentioned, the observed association between high-risk treatment type and rapid recurrence does *not* indicate a causal link between treatment type and poor outcomes, but rather indicates that many patients with aggressive and higher-stage disease, who are more commonly treated with these high-risk modalities, have rapid and aggressive recurrence leading to short survival, regardless of treatment in the definitive or R/M setting.

In conclusion, this nationwide retrospective observational study confirms that the majority of recurrences of both HPV+ and HPV- HNSCC occur within 2 years of definitive therapy. Relapse occurs most rapidly in those with high-risk disease treated with primary chemoradiation and trimodality therapy, who also have significantly shorter OS after initiating R/M therapy compared to those treated with lower-risk modalities. These findings highlight the need for improved treatment strategies both in the definitive and recurrent/metastatic settings for patients with locally advanced HNSCC, as well as implications for clinical trial designs that currently exclude a significant proportion of high-risk patients with progression within 6 months.

## Data Availability

The data analyzed in this study is subject to the following licenses/restrictions: Access to Flatiron Health Data is requested by researcher by submitting data access proposal. Requests to access these datasets should be directed to https://flatironhealth.co.uk/data-governance.

## References

[1] Sung H Ferlay J Siegel RL Laversanne M Soerjomataram I Jemal A . Global cancer statistics 2020: GLOBOCAN estimates of incidence and mortality worldwide for 36 cancers in 185 countries. CA Cancer J Clin. (2021) 71:209–49. doi: 10.3322/caac.21660, PMID: 33538338

[2] Gandhi AK Roy S Thakar A Sharma A Mohanti BK . Symptom burden and quality of life in advanced head and neck cancer patients: AIIMS study of 100 patients. Indian J Palliat Care. (2014) 20:189–93. doi: 10.4103/0973-1075.138389, PMID: 25191005 PMC4154165

[3] SEER . SEER 22 cancer stat facts: oral cavity and pharynx cancer. Rep Cancer. (2024). Available online at: https://seer.cancer.gov/statfacts/html/oralcav.html (Accessed June 16, 2025).

[4] Anderson G Ebadi M Vo K Novak J Govindarajan A Amini A . An updated review on head and neck cancer treatment with radiation therapy. Cancers (Basel). (2021) 13. doi: 10.3390/cancers13194912, PMID: 34638398 PMC8508236

[5] Chang JH Wu CC Yuan KS Wu ATH Wu SY . Locoregionally recurrent head and neck squamous cell carcinoma: incidence, survival, prognostic factors, and treatment outcomes. Oncotarget. (2017) 8:55600–12. doi: 10.18632/oncotarget.16340, PMID: 28903447 PMC5589686

[6] Leeman JE Li JG Pei X Venigalla P Zumsteg ZS Katsoulakis E . Patterns of treatment failure and postrecurrence outcomes among patients with locally advanced head and neck squamous cell carcinoma after chemoradiotherapy using modern radiation techniques. JAMA Oncol. (2017) 3:1487–94. doi: 10.1001/jamaoncol.2017.0973, PMID: 28542679 PMC5710194

[7] Haring CT Kana LA Dermody SM Brummel C McHugh JB Casper KA . Patterns of recurrence in head and neck squamous cell carcinoma to inform personalized surveillance protocols. Cancer. (2023) 129:2817–27. doi: 10.1002/cncr.34823, PMID: 37162461

[8] Pitakpaiboonkul P Jiarpinitnun C Pattaranutaporn P Ngamphaiboon N . Early recurrence, time-to-recurrence, and recurrence patterns: Assessing their impact on survival outcomes in head and neck squamous cell carcinoma (R/M-HNSCC) patients treated with first line platinum-based chemotherapy. Cancer Med. (2024) 13:e7047. doi: 10.1002/cam4.7047, PMID: 38457195 PMC10922020

[9] Oksuz DC Prestwich RJ Carey B Wilson S Senocak MS Choudhury A . Recurrence patterns of locally advanced head and neck squamous cell carcinoma after 3D conformal (chemo)-radiotherapy. Radiat Oncol. (2011) 6:54. doi: 10.1186/1748-717X-6-54, PMID: 21609453 PMC3127781

[10] Larkins E Blumenthal GM Yuan W He K Sridhara R Subramaniam S . FDA approval summary: pembrolizumab for the treatment of recurrent or metastatic head and neck squamous cell carcinoma with disease progression on or after platinum-containing chemotherapy. Oncologist. (2017) 22:873–8. doi: 10.1634/theoncologist.2016-0496, PMID: 28533473 PMC5507654

[11] Black CM Hanna GJ Wang L Ramakrishnan K Goto D Turzhitsky V . Real-world treatment patterns and outcomes among individuals receiving first-line pembrolizumab therapy for recurrent/metastatic head and neck squamous cell carcinoma. Front Oncol. (2023) 13:1160144. doi: 10.3389/fonc.2023.1160144, PMID: 37284189 PMC10241070

[12] Mehanna H Kong A Ahmed SK . Recurrent head and neck cancer: United Kingdom National Multidisciplinary Guidelines. J Laryngol Otol. (2016) 130:S181–s190. doi: 10.1017/S002221511600061X, PMID: 27841130 PMC4873924

[13] Burtness B Harrington KJ Greil R Soulières D Tahara M de Castro G . Pembrolizumab alone or with chemotherapy versus cetuximab with chemotherapy for recurrent or metastatic squamous cell carcinoma of the head and neck (KEYNOTE-048): a randomized, open-label, phase 3 study. Lancet. (2019) 394:1915–28. doi: 10.1016/S0140-6736(19)32591-7, PMID: 31679945

[14] Ma X Long L Moon S Adamson BJS Baxi SS . Comparison of population characteristics in real-world clinical oncology databases in the US: flatiron health, SEER, and NPCR. medRxiv. (2023).

[15] Birnbaum B Nussbaum N Seidl-Rathkopf K Agrawal M Estevez M Estola E . Model-assisted cohort selection with bias analysis for generating large-scale cohorts from the EHR for oncology research. arXiv preprint arXiv:200109765. (2020).

[16] Stalker M Qu K Hwang W-T Cohen RB Mamtani R Sun L . Off-label use of checkpoint inhibitor (CPI) monotherapy in PD-L1–negative or unknown recurrent/metastatic head and neck cancer (R/M HNSCC). J Clin Oncol. (2024) 42:6025–5. doi: 10.1200/JCO.2024.42.16_suppl.6025, PMID: 41735675

[17] Castellanos EH Wittmershaus BK Chandwani S . Raising the bar for real-world data in oncology: approaches to quality across multiple dimensions. JCO Clin Cancer Informa. (2024) 2024:e2300046. 10.1200/CCI.23.00046 PMC10807898 38241599

[18] Yu M Tatalovich Z Gibson JT Cronin KA . Using a composite index of socioeconomic status to investigate health disparities while protecting the confidentiality of cancer registry data. Cancer Causes Contr. (2014) 25:81–92. doi: 10.1007/s10552-013-0310-1, PMID: 24178398

[19] Yost K Perkins C Cohen R Morris C Wright W . Socioeconomic status and breast cancer incidence in California for different race/ethnic groups. Cancer Causes Contr. (2001) 12:703–11. doi: 10.1023/A:1011240019516, PMID: 11562110

[20] NCCN . NCCN clinical practice guidelines in oncology. Head Neck Cancers. (2024) 2024.

[21] Trosman SJ Koyfman SA Ward MC Al-Khudari S Nwizu T Greskovich JF . Effect of human papillomavirus on patterns of distant metastatic failure in oropharyngeal squamous cell carcinoma treated with chemoradiotherapy. JAMA Otolaryngol Head Neck Surg. (2015) 141:457–62. doi: 10.1001/jamaoto.2015.136, PMID: 25742025

[22] Guo T Qualliotine JR Ha PK Califano JA Kim Y Saunders JR . Surgical salvage improves overall survival for patients with HPV-positive and HPV-negative recurrent locoregional and distant metastatic oropharyngeal cancer. Cancer. (2015) 121:1977–84. doi: 10.1002/cncr.29323, PMID: 25782027 PMC4457566

[23] Huang SH Perez-Ordonez B Weinreb I Hope A Massey C Waldron JN . Natural course of distant metastases following radiotherapy or chemoradiotherapy in HPV-related oropharyngeal cancer. Oral Oncol. (2013) 49:79–85. doi: 10.1016/j.oraloncology.2012.07.015, PMID: 22917550

[24] Du E Mazul AL Farquhar D Brennan P Anantharaman D Abedi-Ardekani B . Long-term survival in head and neck cancer: impact of site, stage, smoking, and human papillomavirus status. Laryngosc. (2019) 129:2506–13. doi: 10.1002/lary.27807, PMID: 30637762 PMC6907689

[25] Li H Torabi SJ Yarbrough WG Mehra S Osborn HA Judson B . Association of human papillomavirus status at head and neck carcinoma subsites with overall survival. JAMA Otolaryngology–Head Neck Surg. (2018) 144:519–25. doi: 10.1001/jamaoto.2018.0395, PMID: 29801040 PMC6583856

[26] Sinha P Thorstad WT Nussenbaum B Haughey BH Adkins DR Kallogjeri D . Distant metastasis in p16-positive oropharyngeal squamous cell carcinoma: a critical analysis of patterns and outcomes. Oral Oncol. (2014) 50:45–51. doi: 10.1016/j.oraloncology.2013.10.007, PMID: 24211084 PMC3942323

[27] Fakhry C Zhang Q Nguyen-Tan PF Rosenthal D El-Naggar A Garden AS . Human papillomavirus and overall survival after progression of oropharyngeal squamous cell carcinoma. J Clin Oncol. (2014) 32:3365–73. doi: 10.1200/JCO.2014.55.1937, PMID: 24958820 PMC4195851

[28] Grünwald V Chirovsky D Cheung WY Bertolini F Ahn MJ Yang MH . Global treatment patterns and outcomes among patients with recurrent and/or metastatic head and neck squamous cell carcinoma: Results of the GLANCE H&N study. Oral Oncol. (2020) 102:104526. 31978755 10.1016/j.oraloncology.2019.104526

[29] Gillison ML Trotti AM Harris J Eisbruch A Harari PM Adelstein DJ . Radiotherapy plus cetuximab or cisplatin in human papillomavirus-positive oropharyngeal cancer (NRG Oncology RTOG 1016): a randomized, multicenter, non-inferiority trial. Lancet. (2019) 393:40–50. doi: 10.1016/S0140-6736(18)32779-X, PMID: 30449625 PMC6541928

[30] Vermorken JB Mesia R Rivera F Remenar E Kawecki A Rottey S . Platinum-based chemotherapy plus cetuximab in head and neck cancer. New Engl J Med. (2008) 359:1116–27. doi: 10.1056/NEJMoa0802656, PMID: 18784101

