# Supplementary material for: Recurrent/metastatic relapse after definitive treatment for HNSCC: timing, patterns, and survival
Source: Front Oncol. 2026 Mar 11;16:1738860. doi: 10.3389/fonc.2026.1738860 (PMC13013040; doi:10.3389/fonc.2026.1738860)
Supplement: Supplementary file 1 [file DataSheet1.docx]

**SUPPLEMENTAL FIGURES**

**Supplemental Figure 1. Distribution of cancer stage at diagnosis over type of definitive treatment**


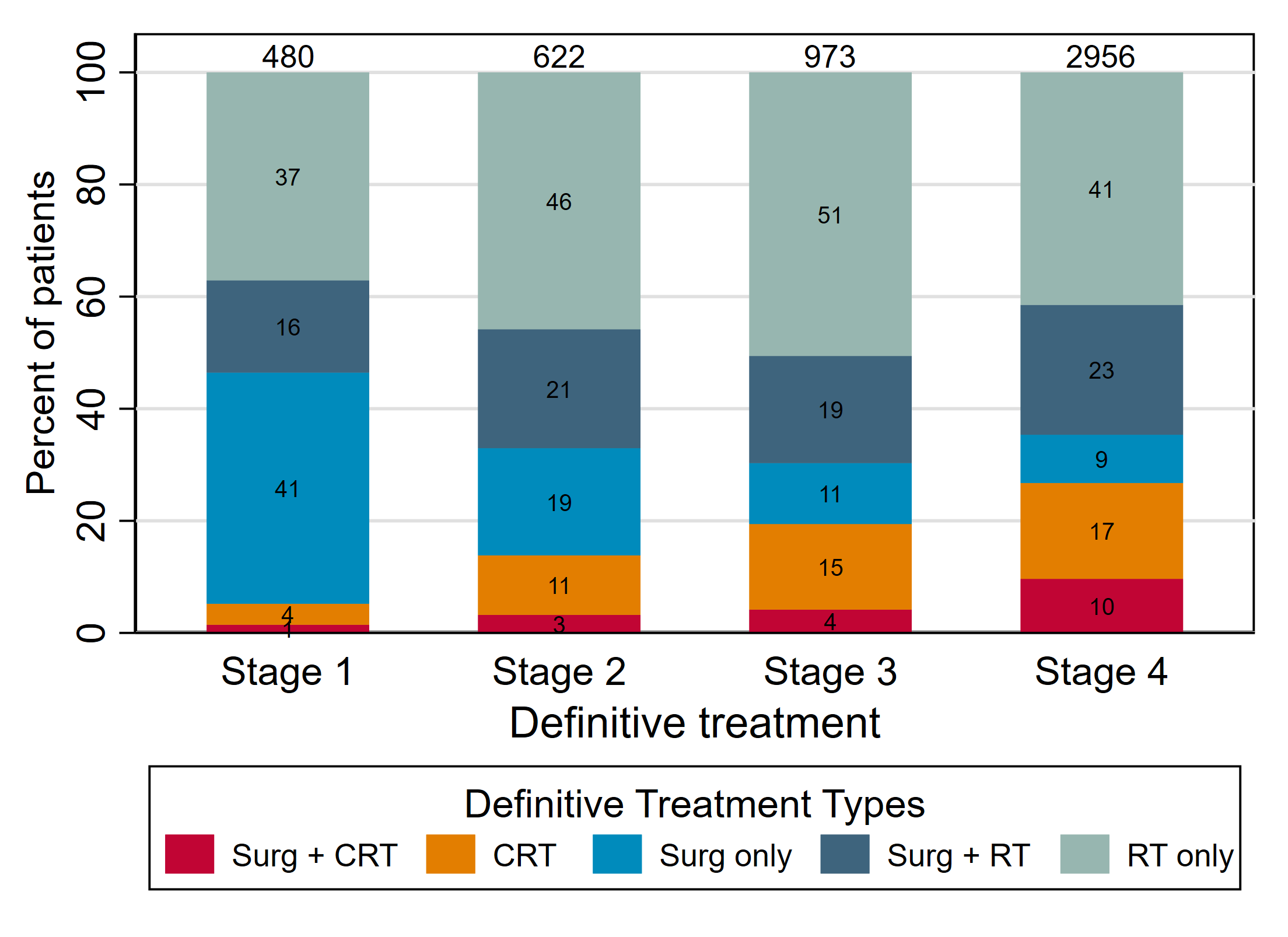


Foot note: Numbers above each bar indicate total numbers of treated patients. Numbers on each bar indicate the percentages for the corresponding treatments.

**Supplemental Figure 2. Distribution of type of treatment over categorized month from definitive treatment**

**
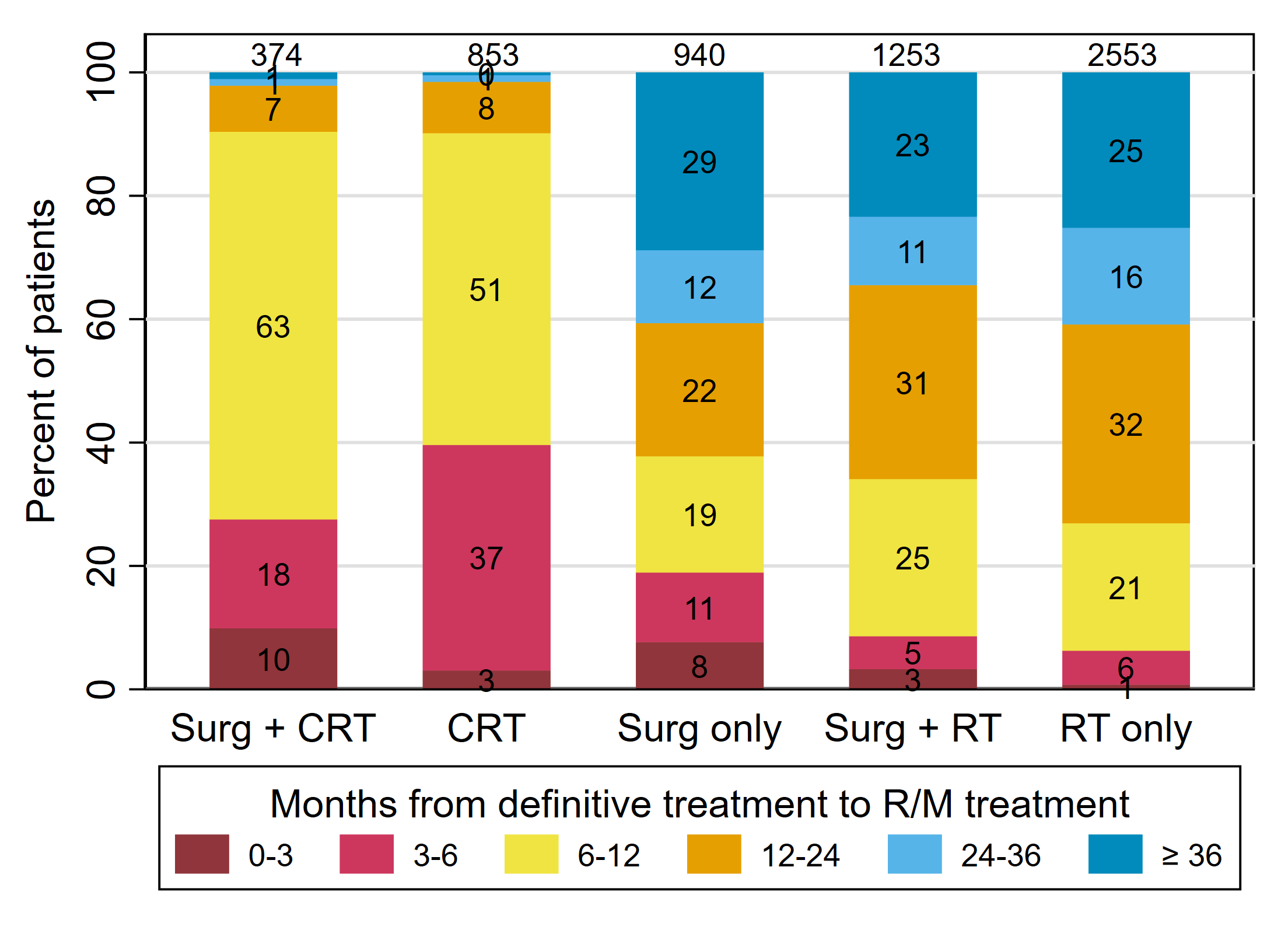
**

Foot note: Numbers above each bar indicate total numbers of treated patients. Numbers on each bar indicate the percentages for the corresponding treatments.

**SUPPLEMENTAL TABLES**

**Supplemental Table 1. Demographics for patients of the full cohort and cohorts by treatment category.**

|  | **Total** | **Surg + CRT** | **CRT** | **Surg only** | **Surg + RT** | **RT only** | **No defn tx** |
| --- | --- | --- | --- | --- | --- | --- | --- |
|  | **N=7,657** | **N=374** | **N=853** | **N=940** | **N=1,253** | **N=2,553** | **N=1,684** |
| Age | 65 (58-72) | 62 (55-69) | 64 (57-71) | 66 (58-74) | 64 (57-71) | 66 (59-73) | 65 (58-72) |
| Sex |  |  |  |  |  |  |  |
| Female | 1,728 (22.6%) | 101 (27.0%) | 174 (20.4%) | 340 (36.2%) | 323 (25.8%) | 459 (18.0%) | 331 (19.7%) |
| Male | 5,929 (77.4%) | 273 (73.0%) | 679 (79.6%) | 600 (63.8%) | 930 (74.2%) | 2,094 (82.0%) | 1,353 (80.3%) |
| Race |  |  |  |  |  |  |  |
| White | 5,130 (73.9%) | 251 (74.7%) | 547 (70.6%) | 670 (78.5%) | 893 (77.4%) | 1,691 (72.4%) | 1,078 (72.5%) |
| Black/AA | 482 (6.9%) | 20 (6.0%) | 67 (8.6%) | 34 (4.0%) | 53 (4.6%) | 174 (7.4%) | 134 (9.0%) |
| Asian | 96 (1.4%) | 5 (1.5%) | 10 (1.3%) | 18 (2.1%) | 34 (2.9%) | 12 (0.5%) | 17 (1.1%) |
| Other | 1,234 (17.8%) | 60 (17.9%) | 151 (19.5%) | 131 (15.4%) | 174 (15.1%) | 460 (19.7%) | 258 (17.4%) |
| Missing | 715 (-) | 38 (-) | 78 (-) | 87 (-) | 99 (-) | 216 (-) | 197 (-) |
| Smoking status |  |  |  |  |  |  |  |
| No smoking history | 1,639 (21.5%) | 95 (25.5%) | 151 (17.7%) | 278 (29.6%) | 336 (26.9%) | 487 (19.2%) | 292 (17.4%) |
| Current or former smoker | 5,983 (78.5%) | 278 (74.5%) | 701 (82.3%) | 661 (70.4%) | 912 (73.1%) | 2,049 (80.8%) | 1,382 (82.6%) |
| Missing | 35 (-) | 1 (-) | 1 (-) | 1 (-) | 5 (-) | 17 (-) | 10 (-) |
| PD-L1 level |  |  |  |  |  |  |  |
| CPS ≥ 20 | 852 (37.7%) | 56 (44.8%) | 84 (37.0%) | 130 (50.4%) | 163 (40.9%) | 259 (32.8%) | 160 (34.6%) |
| CPS 1-19 ^a^ | 921 (40.8%) | 46 (36.8%) | 96 (42.3%) | 92 (35.7%) | 152 (38.1%) | 327 (41.4%) | 208 (45.0%) |
| CPS <1 | 487 (21.5%) | 23 (18.4%) | 47 (20.7%) | 36 (14.0%) | 84 (21.1%) | 203 (25.7%) | 94 (20.3%) |
| Unknown ^b^ | 5,397 (-) | 249 (-) | 626 (-) | 682 (-) | 854 (-) | 1,764 (-) | 1,222 (-) |
| ECOG PS (ref: 0-1) |  |  |  |  |  |  |  |
| 0-1 | 5,029 (79.4%) | 244 (76.5%) | 547 (74.5%) | 624 (79.9%) | 836 (80.9%) | 1,726 (81.3%) | 1,052 (78.4%) |
| ≥ 2 | 1,304 (20.6%) | 75 (23.5%) | 187 (25.5%) | 157 (20.1%) | 197 (19.1%) | 398 (18.7%) | 290 (21.6%) |
| Missing | 1,324 (-) | 55 (-) | 119 (-) | 159 (-) | 220 (-) | 429 (-) | 342 (-) |
| Primary site |  |  |  |  |  |  |  |
| HPV+ Oropharynx | 1,928 (25.8%) | 50 (13.6%) | 233 (28.1%) | 79 (8.5%) | 248 (20.3%) | 890 (35.5%) | 428 (26.6%) |
| HPV- Oropharynx | 1,581 (21.2%) | 55 (15.0%) | 244 (29.4%) | 96 (10.3%) | 185 (15.2%) | 561 (22.4%) | 440 (27.4%) |
| Larynx | 1,567 (21.0%) | 48 (13.1%) | 170 (20.5%) | 101 (10.9%) | 208 (17.0%) | 696 (27.8%) | 344 (21.4%) |
| Oral Cavity | 1,899 (25.4%) | 200 (54.5%) | 93 (11.2%) | 633 (68.1%) | 539 (44.1%) | 205 (8.2%) | 229 (14.2%) |
| Hypopharynx | 487 (6.5%) | 14 (3.8%) | 89 (10.7%) | 21 (2.3%) | 41 (3.4%) | 155 (6.2%) | 167 (10.4%) |
| Unknown | 195 (-) | 7 (-) | 24 (-) | 10 (-) | 32 (-) | 46 (-) | 76 (-) |
| Patterns of recurrence |  |  |  |  |  |  |  |
| Local only | 1,473 (27.1%) | 5 (2.0%) | 7 (1.2%) | 400 (46.5%) | 351 (30.6%) | 663 (27.4%) | 47 (24.1%) |
| Distant only | 3,198 (58.9%) | 245 (98.0%) | 555 (98.6%) | 213 (24.8%) | 633 (55.2%) | 1,423 (58.8%) | 129 (66.2%) |
| Local + distant | 762 (14.0%) | 0 (0.0%) | 1 (0.2%) | 247 (28.7%) | 162 (14.1%) | 333 (13.8%) | 19 (9.7%) |
| Not recorded | 2,224 (-) | 124 (-) | 290 (-) | 80 (-) | 107 (-) | 134 (-) | 1,489 (-) |
| Year of treatment |  |  |  |  |  |  |  |
| 2011-2018 | 4,262 (55.7%) | 177 (47.3%) | 459 (53.8%) | 517 (55.0%) | 729 (58.2%) | 1,393 (54.6%) | 987 (58.6%) |
| 2019 | 765 (10.0%) | 30 (8.0%) | 96 (11.3%) | 95 (10.1%) | 110 (8.8%) | 263 (10.3%) | 171 (10.2%) |
| 2020 | 735 (9.6%) | 42 (11.2%) | 86 (10.1%) | 105 (11.2%) | 111 (8.9%) | 256 (10.0%) | 135 (8.0%) |
| 2021 | 736 (9.6%) | 50 (13.4%) | 71 (8.3%) | 95 (10.1%) | 99 (7.9%) | 241 (9.4%) | 180 (10.7%) |
| 2022 | 725 (9.5%) | 47 (12.6%) | 92 (10.8%) | 74 (7.9%) | 122 (9.7%) | 255 (10.0%) | 135 (8.0%) |
| 2023 | 434 (5.7%) | 28 (7.5%) | 49 (5.7%) | 54 (5.7%) | 82 (6.5%) | 145 (5.7%) | 76 (4.5%) |
| SES ^c^ | 3 (2-4) | 3 (2-4) | 3 (2-4) | 3 (2-4) | 3 (2-4) | 3 (2-4) | 3 (2-4) |
| Practice type |  |  |  |  |  |  |  |
| Community | 5,838 (76.2%) | 253 (67.6%) | 712 (83.5%) | 656 (69.8%) | 842 (67.2%) | 1,972 (77.2%) | 1,403 (83.3%) |
| Academic | 1,819 (23.8%) | 121 (32.4%) | 141 (16.5%) | 284 (30.2%) | 411 (32.8%) | 581 (22.8%) | 281 (16.7%) |
| Frontline treatment |  |  |  |  |  |  |  |
| Chemotherapy +/- cetuximab | 4,675 (61.1%) | 167 (44.7%) | 381 (44.7%) | 707 (75.2%) | 741 (59.1%) | 1,359 (53.2%) | 1,320 (78.4%) |
| CPI with chemotherapy | 864 (11.3%) | 53 (14.2%) | 83 (9.7%) | 68 (7.2%) | 163 (13.0%) | 297 (11.6%) | 200 (11.9%) |
| CPI monotherapy | 2,118 (27.7%) | 154 (41.2%) | 389 (45.6%) | 165 (17.6%) | 349 (27.9%) | 897 (35.1%) | 164 (9.7%) |

Data are presented as median (IQR) for continuous measures, and n (%) for categorical measures.

For categorical variables which include missing/unknown, percentages are out of known categories and missing/unknown are listed without %.

a. CPS 1-19 includes “positive, not otherwise specified” in 41, 1, 2, 5, 9, 12, and 12 patients with total, Surg + CRT, CRT, Surg only, Surg + RT, RT only, and no definitive treatment groups, respectively.

b. Unknown includes 200, 13, 30, 16, 34, 68, and 39 patients with total, Surg + CRT, CRT, Surg only, Surg + RT, RT only, and no definitive treatment groups, respectively, with PD-L1 test attempted but no result available.

c. 5-level indicator of neighborhood socioeconomic conditions (1 - lowest SES; 5 - highest SES)

Abbreviations: Oropharyngeal squamous cell carcinoma (OPSCC), combined positive score (CPS), ECOG performance status (ECOG PS), surgery (Surg), chemoradiation (CRT), radiation (RT), definitive treatment (defn tx), socioeconomical status (SES).
